# Flexible Pressure Sensor with Ag Wrinkled Electrodes Based on PDMS Substrate

**DOI:** 10.3390/s16122131

**Published:** 2016-12-14

**Authors:** Jianli Cui, Binzhen Zhang, Junping Duan, Hao Guo, Jun Tang

**Affiliations:** Science and Technology on Electronic Test & Measurement Laboratory, North University of China, Taiyuan 030051, China; zhaorui@nuc.edu.cn (J.C.); duanjunping@nuc.edu.cn (J.D.); zhaorui2012@pku.edu.cn (H.G.)

**Keywords:** pressure sensor, flexible sensor, Ag wrinkled electrodes, carbon nanotube

## Abstract

Flexible pressure sensors are essential components of electronic skins for future attractive applications ranging from human healthcare monitoring to biomedical diagnostics, robotic skins, and prosthetic limbs. Here we report a new kind of flexible pressure sensor. The sensors are capacitive, and composed of two Ag wrinkled electrodes separated by a carbon nanotubes (CNTs)/polydimethylsiloxane (PDMS) composite deformable dielectric layer. Ag wrinkled electrodes were formed by vacuum deposition on top of pre-strained and relaxed PDMS substrates which were treated using an O_2_ plasma, a surface functionalization process, and a magnetron sputtering process. Ultimately, the developed sensor exhibits a maximum sensitivity of 19.80% kPa^−1^ to capacitance, great durability over 500 cycles, and rapid mechanical responses (<200 ms). We also demonstrate that our sensor can be used to effectively detect the location and distribution of finger pressure.

## 1. Introduction

As a key component in the next generation of flexible electronics, flexible pressure sensors have drawn more attention in human-oriented future technologies such as electronic skins [1,2], wearable healthcare monitors [3,4], robotic skins [5], and touch interfaces [6] in recent years. In the meantime, much effort has been made toward improving the sensitivity of a flexible pressure sensor in the range of less than 10 kPa to realize the most of the aforementioned technologies that mimic human skin or human tactile receptors. A variety of pressure-sensing technologies have been categorized in these patterns, including piezoelectric [7], piezoresistive [8], and capacitive-types [9,10]. No matter which sensory model is chosen, those with high sensitivity and flexibility and low cost are desirable in this field.

Up to now, various nanomaterials, including nanowires [11,12], carbon nanotubes [13,14], polymer nanofibers [15,16], metal nanoparticles [17], and graphene [18] have been used for the design of novel flexible pressure sensors. To solve the problem of the poor sensitivity of the flexible pressure sensors, some methods of introducing micro-or nano-structures on the surface of a thin dielectric layer (e.g., micro-pyramids and nano-needles [19]) have been suggested. In addition, various metals have been used as flexible substrate for capacitive pressure sensors [20,21], including polyethylene terephthalate, indium tin oxides, and poly(3,4-ethylenedioxythiophene). However, the proposed techniques face similar problems: the fabrication of complicated micro/nano-structures, challenging scalability, high cost, demanding materials, and poor adhesion between the metal materials and the substrate.

Herein, we demonstrate a simple process for the development of a flexible capacitive pressure sensor based on ultrasensitive carbon nanotubes/polydimethylsiloxane (CNTs/PDMS) composite elastomers dielectric layer and Ag wrinkled electrodes on the PDMS substrate. In order to enhance the adhesion between the metal materials and the substrate, we have followed a combined approach to the surface modification of the PDMS surface by both O_2_ plasma and sodium dodecyl sulfate (SDS) solution. The constructed flexible pressure sensor will be characterized by its mechanical-capacitance response with different sizes of the sensing area and applied loading values. The result shows that the sensor presents high sensitivity, rapid mechanical response, a large working pressure range, and great durability and repeatability. In addition, this pressure sensor will demonstrate the capability to effectively detect the location and distribution of finger pressure. Moreover, the entire preparation and fabrication process of such a pressure sensor is easy to fabricate and compatible with conventional micro/nanofabrication technology, which permits scalable production at a significantly lower unit-cost.

## 2. Experimental

### 2.1. Material Preparation

For the preparation of CNTs/PDMS nanocomposites, first the hydrocarbonyl CNTs (diameter = 10–20 nm, length = 0.5–2 μm, purity > 95%, CheapTubes Inc., Cambridge, MA, USA) and absolute ethanol were homogenized by sonication at 30 °C for 2 h to obtain well-dispersed CNTs suspension. Second, a PDMS base (Sylgard 184, Dow Corning, Midland, MI, USA), a 1:40 ratio for curing agent to base) was added to the above mixture and blended using a glass rod for 15 min, and then heated on the baking at 150 °C until almost all of the absolute ethanol was evaporated. After cooling, air bubbles in the mixture were removed under mild vacuum for 1 h. For the preparation of the dielectric layer, the as-prepared CNTs/PDMS mixture was transferred to a square mold (8 × 8 mm^2^ × 800 μm) and moved into a vacuum chamber for 2.5 h at 70 °C. Finally, the cured square CNTs/PDMS composite with a size of 8 mm × 8 mm × 800 μm was obtained.

### 2.2. Device Fabrication

**Figure 1:**
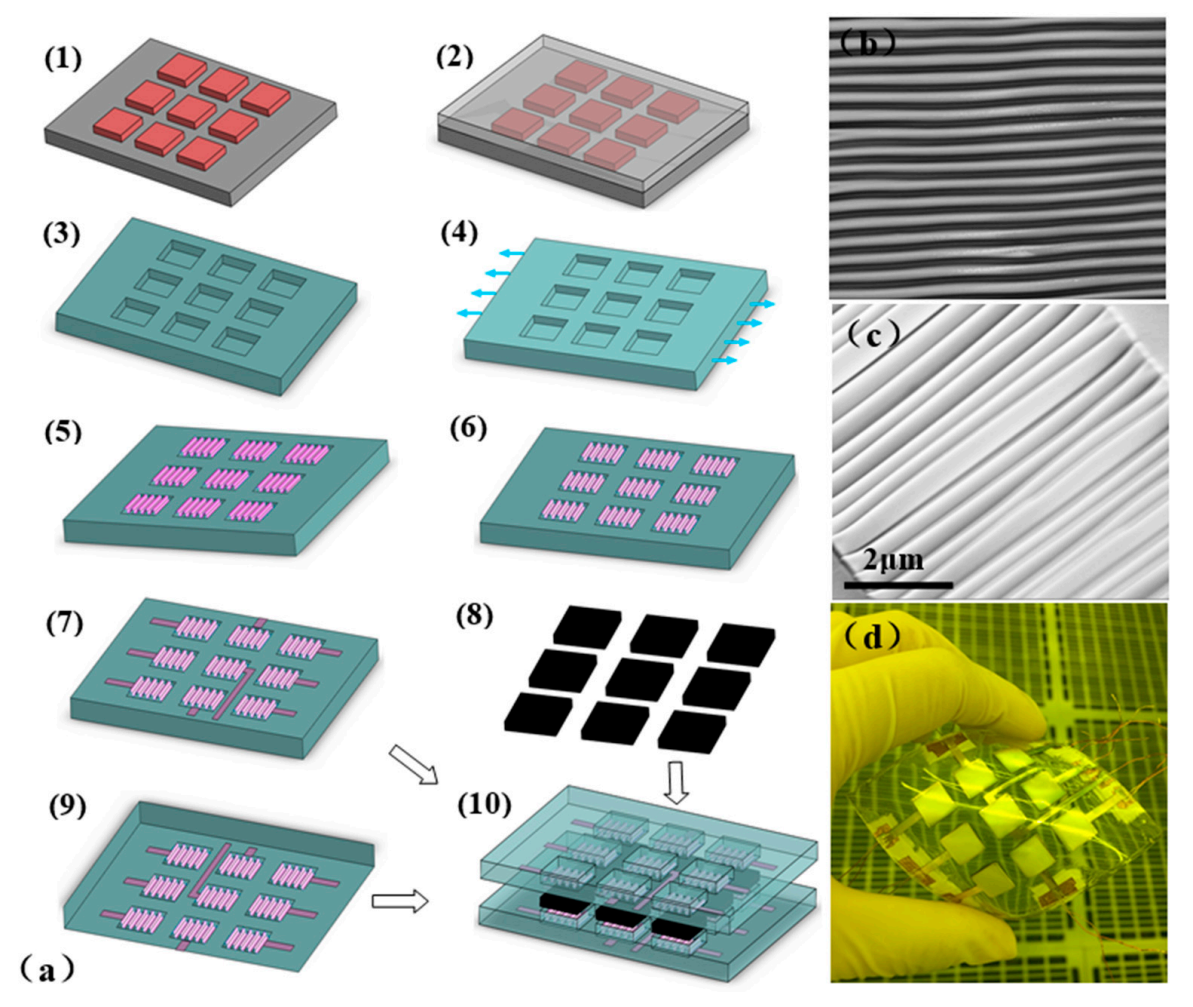
(**a**) Schematic diagrams of the fabrication procedure for the flexible pressure sensor: (1) SU-8 mold; (2) Polydimethylsiloxane (PDMS) casting; (3) PDMS mold; (4) Pre-strain PDMS; (5) Pre-strain relax after O_2_ plasma treatment; (6) Sodium dodecyl sulfate (SDS) surface functionalization; (7) Ag wrinkled electrodes on the PDMS substrate after Ag sputtering; (8) Carbon nanotubes (CNTs)/PDMS elastomer dielectric layer; (9) Ag wrinkled electrodes on the PDMS substrate; (10) Flexible pressure sensor; (**b**) Laser confocal image of the electrode pattern; (**c**) Cross-section of the fabricated electrode from an SEM image; (**d**) digital image of a typical flexible pressure sensor with 3 × 3 detecting units.

The ultra-thick SU-8 UV photolithography process adopted in this paper is similar to the one reported in [22,23]. A 4-inch Si wafer was cleaned using acetone, isopropyl alcohol (IPA), and deionized water, sequentially. After dehydration at 180 °C for about 20 min, SU-8 100 photoresist was spin-coated on the silicon wafer at 800 rpm for 30 s to obtain a 400 μm thick SU-8 layer. It was then soft-baked on a well-leveled hot plate for 1 h at 65 °C and 4 h at 95 °C. In order to reduce internal stress in the thick SU-8 layer, a soft bake process consisting of multiple ramping and dwelling steps was used. The wafer was exposed to an EVG-610 mask aligner for a dose of 3 × 500 mJ/cm^2^ with a 30 s interval. A post-bake was performed in controlled temperature slope to form a strong crosslink. Finally, the wafer was immersed into SU-8 developer for development to obtain the SU-8 mold with a thickness of 400 μm, as in Figure 1(a1).

For the preparation of the stretchable Ag electrodes, first a PDMS prepolymer was prepared with a Sylgard 184:curing agent weight ratio of 10:1. The PDMS prepolymer was then degassed in a vacuum desiccator for 15 min to remove air bubbles. The degassed PDMS prepolymer was poured onto the SU-8 mold (with a thickness of 600 μm attained by controlling the spinning speed) and degassed again. Thirdly, it was cured at 70 °C for 3 h. Finally, the PDMS layer was carefully peeled off the SU-8 master mold. The PDMS layer (as in Figure 1(a3)) will be used as the substrate of the stretchable Ag electrodes process.

As illustrated in Figure 1(a4), the PDMS film was firstly pre-strained up to 160% [24] by utilizing a self-made clamp. Then oxygen plasma treatment was used to modify the hydrophobic PDMS with hydrophilic functionalities. A SiOx layer and hydrophilic groups (e.g., –OH) were thus formed on pre-strained PDMS substrates by the O_2_ plasma, as reported in our previous work [25]. When the strain of PDMS exceeds a critical value, the PDMS film self-assembles to form folded grating structures after strain relaxation [26], as in Figure 1(a5). What you should note here is that only the grating structures on the square plates (8 × 8 mm^2^) was described. The PDMS films were then immersed in an SDS solution with concentration of 1% for 60 s to introduce –SO_3_^−^ groups at the surface of the wrinkled PDMS grating, which can ensure a tight contact between Ag+ and PDMS through condensation reactions of hydrophilic functionalities. Finally, as shown in Figure 1(a7), an Ag film was coated on the PDMS films surface via a magnetron sputtering process (60 W, 8.0 × 10^−3^ Torr) to obtain the Ag wrinkled electrodes based on PDMS substrate. Meanwhile, a metal mask layer was used to produce the electrode-lead in this process. As illustrated in Figure 1(a10), the pressure sensors were fabricated by pouring CNTs/PDMS dielectric layer at the middle of the two layers of the parallel-plate capacitor cavity with a Ag wrinkled electrodes on the PDMS substrate and curing at 95 °C for 2 h to complete hot-press bonding and obtain the capacitive pressure sensor. Finally, each electrode was connected with copper wires using silver paste. More details can be seen in Figure 1a. A typical flexible pressure sensor with 3 × 3 detecting units is shown in Figure 1d.

Figure 1b shows the optical image obtained using a Laser Confocal Microscope (OLS4100, Olympus, Tokyo, Japan), the wrinkled electrode structure has sine-like periodic grating structures. The sine-shaped grating can be stretched in the grid line direction without tearing, and consequently, this is an important mechanism for the manufacture of electrode structures of the stretchable capacitor. The SEM image in Figure 1c shows that the wrinkled electrode structure has a smooth surface, uniform lines, and a period of about 1 μm.

## 3. Results and Discussion

### 3.1. Material Preparation

**Figure 2:**
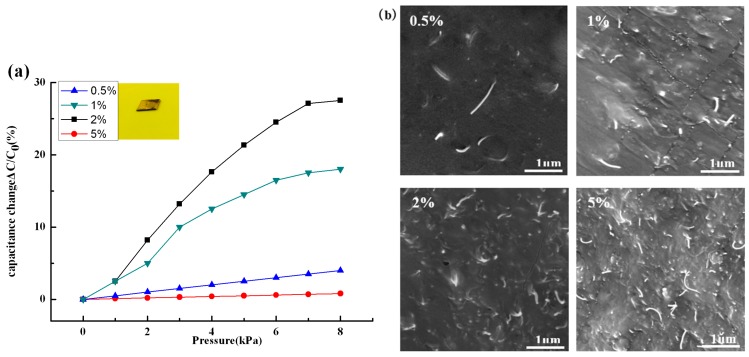
Characterization of different volume fraction of CNTs/PDMS elastomers. (**a**) Mechanical capacitance response of square CNTs/PDMS film (8 mm × 8 mm × 0.5 mm); (**b**) SEM images of CNTs/PDMS elastomers with different CNT concentrations.

As mentioned in [27], material sensitivity plays a vital role in optimal device performance. Herein, CNTs were used to prepare PDMS-based composite elastomers and dielectric layer. Microscopic analysis was used to analyze these results in Figure 2b. It can be found that CNTs have a uniform dispersion in the PDMS matrix, and even a single CNT can be distinguished. The results also show that the amount of CNTs increased, with its concentration increasing from 0.5%, 1%, 1.5%, and 2%, to 5%.

Mechanical-capacitance response tests of square CNTs/PDMS composite were also investigated to optimize their dynamic capacitance sensitivity, as seen in Figure 2a. In this detecting section, the double-tape conductive copper has been pasted on both sides of the square CNTs/PDMS composite film, and a shielding wire was welded to one side of it, which was used as a connecting link connected to the external measurement device. As is shown in Figure 2a, when the concentration of CNTs is between 0.5% and 3%, the fastest change rate of capacitance can be observed. It shows that the prepared CNTs/PDMS composite with different CNTs concentrations exhibited large capacitance variation under the external loading from 0 Pa to 8 kPa^−1^. Especially when the CNTs concentration is 2%, the results demonstrated their ultrasensitive properties. Specifically, when the external loading is 4 kPa, the capacitance variation is up to 18%. This value is about 1.6 times of that of square CNTs/PDMS film (1.5%, CNTs concentration). The square CNTs/PDMS film exhibits small capacitance variation until the CNTs concentration is up to 5%. The reason is that the distance between the CNTs particles will decrease with increasing CNTs concentration, and once the concentration increased to a certain degree, a connection phenomenon between particles will occur. Meanwhile, the capacitance characteristic will be destroyed between the CNTs particles. This phenomenon demonstrates that the piezocapacitance effect is very crucial in the capacitance variation of the compressed CNTs/PDMS film, as well as in the sensitivity of the final device. Conclusively, a square CNTs/PDMS film with a CNTs concentration of 2% was used in the following test.

### 3.2. Characterization of the Sensitivity

**Figure 3:**
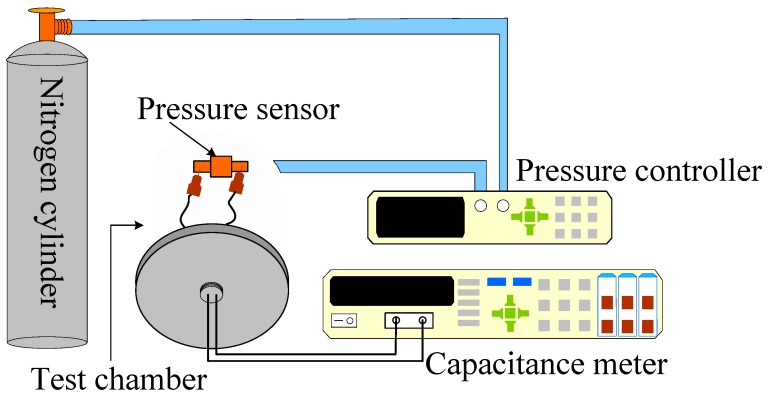
Experimental setup. The sensor was mounted in the pressure chamber. The applied force and capacitance change were measured by the pressure controller and capacitance meter, respectively.

The capacitive pressure sensor was characterized by using the experimental setup shown in Figure 3. Pressure was applied to the capacitive sensor to test its pressure sensitivity. A pressure controller (PACE5000, GE Sensing & Inspection Technologies, Billerica, MA, USA) was used as the pressure application tool. To enhance the stability of the pressure sensor, a conductive copper was used as a connecting link between pressure leads and the impedance analyzer (Agilent 4284A, Santa Clara, CA, USA) during capacitance output testing. An external pressure ranging from 0 to 10 kPa^−1^ was applied, and the capacitance of 2.17 pF at the 0 Pa pressure was defined as the base capacitance C_0_.

**Figure 4:**
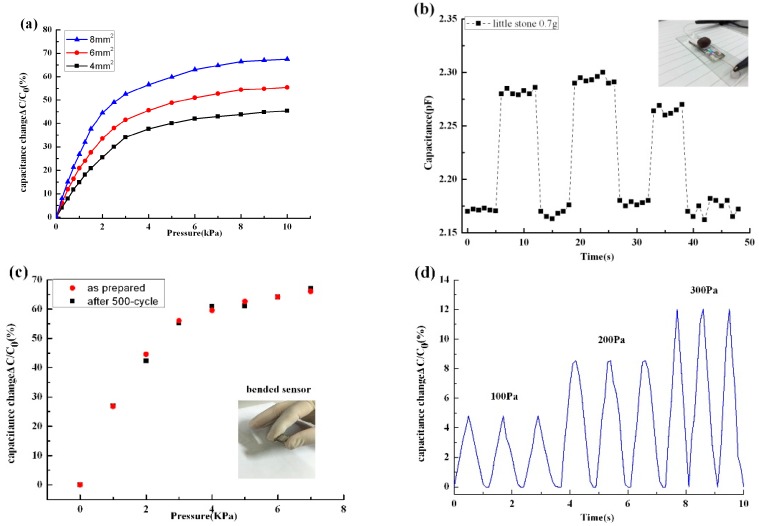

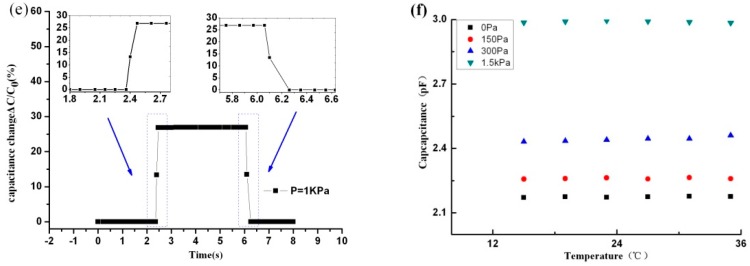
Characterization of the capacitive pressure response of the pressure sensor. (**a**) Sensitivity with different sensing area (size = 4 × 4 mm^2^, 6 × 6 mm^2^, and 8 × 8 mm^2^, respectively); (**b**) Capacitance–time curve for the detection of pressure (100 Pa) according to the loading and unloading of a little stone (0.7 g); (**c**) Bending stability of pressure response after 500-cycle bending test; (**d**) Multi-cycle tests of dynamic loading/unloading pressure with different values; (**e**) Fast response and relaxation time (<200 ms) of the sensor; (**f**) The curve of thermal drift of the sensor.

The pressure sensitivity of the pressure sensor (S) can be defined as the slope of the relative capacitance change–pressure (S = δ(ΔC/C_0_)/δp; ΔC = C − C_0_, where C and C_0_ denote the capacitance without and with applied pressure, respectively) over the external pressure loading (P) [28] as shown in Figure 4a.

The results show that the capacitance variation increased with the increase of the external loading. Meanwhile, sensitive curves with two segments were observed. This phenomenon can be explained by the fact that the distance of the wrinkled electrode between the upper-plate and the bottom-plate of the sensor and the sensitivity of the CNTs/PDMS elastomer dielectric layer will be rapidly increased with the increased pressure; however, the higher pressure will generate a large deformation of the sensing electrode membrane. In the meantime, the high shear force from the edge of the sensing membrane cannot be ignored, leading to the deviation of the device sensitivity from a linear relationship with the external pressure. Moreover, when the size of the sensing area increases from 4 × 4 mm^2^, 6 × 6 mm^2^, to 8 × 8 mm^2^, the sensitivity of the device increases from 11.9% kPa^−1^ to 15.06% kPa^−1^ and 19.80% kPa^−1^, respectively. The deformation of the square membrane with edge clamped is not only up to the flexural rigidity of the membrane itself, but is also in close relationship with the size of the square membrane. So, the large size will bring large deformation of the sensing membrane under the same external loading, showing a high sensitivity. As is illustrated in Figure 4b, the pressure sensor can detect the loading and unloading of a little stone of weight 0.7 g. The pressure of the little stone was about 100 Pa. The capacitance change of the sensor can detect the small pressure.

In addition, the pressure sensor also featured good flexibility because of the highly flexible Ag wrinkled electrodes employed [24], and it could be bent freely in all directions at a very small bending radius of 4 mm, as shown in the inset to Figure 4c. To investigate the reliability of the bending of the flexible sensor, the relative capacitance change–pressure curves of the sensor were measured before bending and after 500 bending cycles. From Figure 4c, it is seen that the relative capacitance changes of the 500 cycles of bending-tested sensor at each pressure show no appreciable degradation compared to the as-prepared sensor. The pressure sensor with the wrinkled-structured electrode is robust and stable at repeated loading/unloading and bending cycles, based on the above results. Figure 4d shows the multi-cycle tests of dynamic loading/unloading pressure with different values of the sensors. The results show that every response profile with different loading cycles (100 Pa, 200 Pa, and 300 Pa, respectively) is very regular, stable, and continuous. Figure 4e and insets show the response and relaxation times of the sensor. When a pressure of 1 kPa^−1^ was loaded and unloaded to the sensor, the response and relaxation times were less than 200 ms. The capacitance variation of the samples have also been investigated against heating using the temperature test chamber with a temperature step of 4 °C (range from 15 °C to 35 °C) under different external loading. As seen in Figure 4f, the capacitance had a small change with the variation of temperature. This effect can be attributed to the thermal expansion coefficient of the PDMS, which results in the micro-thermal deformations of the sensor. However, the change of shape under these environments is much smaller than external loading. From the electrical characterizations, it can also be concluded that the fabricated sensors have sufficient stability and can implement the pressure sensing.

### 3.3. Surface Topology Sensing

**Figure 5:**
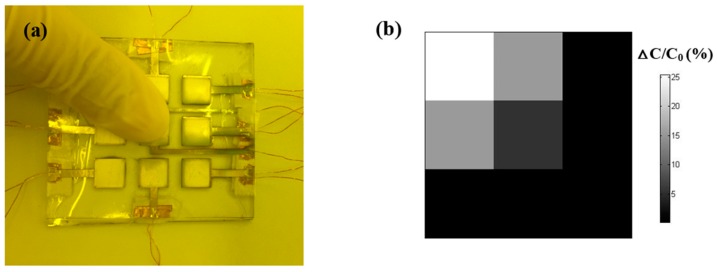
(**a**) Digital image of the fingers on the surface of the pressure sensor to test the finger pressure-sensing capability; (**b**) Finger pressure distribution presented by capacitance variation of every detecting unit.

To demonstrate the efficiency of this device, the flexible pressure sensor with 3 × 3 detecting units was used to map finger pressure (as shown in Figure 5a), in which each detecting unit (8 × 8 mm^2^) can be regarded as a chip-type sensing component. When a finger was loaded on the top of our sensors, the detected pressure signals were recorded and plotted as a color intensity map (Figure 5b). Each square represents the relevant detecting unit. The color intensity corresponds to the capacitance variation of this detecting unit under the external loading. The results show that this device can simultaneously and precisely detect the amplitudes and local pressure distributions in consistency with the finger pressures. This capability is the typical feature of the electronic skin.

## 4. Conclusions

In summary, a new kind of flexible pressure sensor based on Ag wrinkled electrodes and CNTs/PDMS elastomer composite on the PDMS substrate was successfully designed and fabricated. The sensitive mechanism was mainly based on the capacitance variation of Ag wrinkled electrodes between the upper plates and the bottom one and the piezocapacitance effect of CNTs/PDMS elastomer composite. The developed sensor exhibits a maximum sensitivity of 19.80% kPa^−1^, durability over 500 cycles, and rapid mechanical responses (<200 ms) with the defined parameters. Meanwhile, problems such as sensor failure resulting from bending and deformation were effectively solved in basic research owing to the proposed Ag wrinkled electrodes. Our flexible sensor demonstrated its capability to accurately detect and convey the location and distribution of external loading. We believe that the designs and operational principles of the devices present a feasible, facilitative, and robust technology platform for the wide applications of various multifunctional electric devices with optimal performances. Furthermore, these stretchable sensors are easy to fabricate and are compatible with conventional micro/nano fabrication technology, which guarantees the consistency of each detection unit of array sensor according to ultra-thick SU-8 UV photolithography process and is significant for the future application of electronic skins in smart robotic systems.
